# The Value of Admission Serum IL-8 Monitoring and the Correlation with IL-8 (-251A/T) Polymorphism in Critically Ill Patients

**DOI:** 10.1155/2014/494985

**Published:** 2014-03-06

**Authors:** Ayman Abd Al-Maksoud Yousef, Ghada Abdulmomen Suliman, Maaly Mohamed Mabrouk

**Affiliations:** Faculty of Medicine, Tanta University, Tanta, Egypt

## Abstract

*Background*. The clinical management of sepsis is a highly complicated process. Disruption of the immune system explains in part the major variation in sepsis outcome. IL-8 is a proinflammatory cytokine, genetic polymorphism of this cytokine could explain the outcome of sepsis. The present study was conducted to determine the value of serum IL-8 monitoring and its (-251A/T) genetic polymorphism in critically ill patients. *Patients and Methods*. 180 critically ill patients were allocated into two groups, 90 septic patients (sepsis group) and 90 nonseptic patients (SIRS group). Admission serum IL-8 and its (-251A/T) mutant allele were detected. *Results*. The admission mean value of serum IL-8 was significantly elevated in sepsis group. In both groups, the mean value of serum IL-8 in nonsurvived patients and patients with IL-8 (-251A/T) mutant allele was significantly higher. A positive correlation of survival and IL-8 (-251A/T) mutant allele was detected in both groups. The serum IL-8 distinguished wild from IL-8 (-251A/T) mutant allele at a cut-off value of 600 pg/mL. *Conclusion*. The admission mean value of serum IL-8 was significantly elevated in septic, nonsurvived, and patients with IL-8 (-251A/T) mutant alleles. A positive correlation of survival and IL-8 (-251A/T) mutant allele patients was detected.

## 1. Introduction

Sepsis constitutes a complex syndrome in critically ill patients, which usually correlates with bad prognosis; the outcome of sepsis is mostly determined by major interaction between the host, the invading microorganism, and the surrounding environment. Wide variability exists in the susceptibility to and outcome from sepsis even within similar intensive care unit populations. Some of this variability in the host may be due to genetic variation in genes coding for components of the innate immune response.

Genetic association studies suggest a major genetic influence on outcome of sepsis. Dysregulation of innate immunity, especially those genes involved in inflammatory pathway, is expected to be the determinant for manifestation of sepsis [1, 2]. Evaluation of the association between the genetic polymorphism in septic patients provides a new tool to predict prognosis [3]. Interlukine-8 (IL-8) is a proinflammatory cytokine, has a potential role in regulating the innate immune response to bacterial infection, and has become crucial in the management, early prediction, monitoring and success of antimicrobial therapy in critically ill patients [4]. IL-8 belongs to CXC chemokine family, which is the major neutrophil chemo-attractant and activator. Genetic polymorphism in IL-8 at position-251 had been previously studied in various pathological conditions; furthermore, this polymorphism was associated with diseases that include respiratory syncytial virus [5]. So, the present study is conducted to determine the value of serum IL-8 monitoring and the role of IL-8 (-251A/T) polymorphism and its correlation with survival in critically ill patients.

## 2. Materials and Methods

After the study was approved by an Investigational Review Board, an informed consent was obtained from patients participating in the study or their relatives. This study was conducted in the ICU of Emergency Hospital of Tanta University, Tanta, Egypt. It is a 25-bed medical/surgical ICU. A total of one hundred eighty adult intensive care patients (97 men and 83 women) were included in the study. Ninety patients developed septic complication during ICU stay (*sepsis group*). Ninety patients were critically ill without evidence of infectious organism (*SIRS group*). Patients received anti-inflammatory drugs or corticosteroids before admission, patients with immunosuppressive illness, patients with chronic organ failure; patients received massive blood transfusion and patients with radiation therapy or previous organ transplantation were excluded from the study. At admission, the patient's age, sex, height, and weight were recorded, in addition to clinical status; sequential organ failure assessment (*SOFA*) score; temperature; heart rate; respiratory rate; blood pressure; central venous pressure; laboratory analysis (complete blood count, blood urea nitrogen, blood sugar, serum sodium, potassium, calcium, aspartate aminotransferase, alanine aminotransferase, prothrombin time, albumin, and CRP); and arterial blood gas analysis were measured. Routine cultures of blood, urine and suspected sites were obtained to determine the presence of infection. We attempted to maintain the patient hemoglobin level at 10–12 g/dL and central venous pressure at 8–12 cm H_2_O. If needed, blood products, intravascular fluid replacement, and inotropic and/or vasopressor agents were administered. Each day the attending physician in the ICU evaluated all the study patients for sepsis, severe sepsis, or septic shock.

The signs of sepsis were body temperature <36°C or >38°C, tachycardia (>90 beats/min), ventilatory frequency >20 breath/min or PCO_2_ <32 mmHg (unless the patient was mechanically ventilated), a white cell count ≥12 × 10^9^ litre^−1^ or <4 × 10^9^ litre^−1^, or >10% immature neutrophils, in addition to the presence of infection [6]. Severe sepsis was defined as sepsis with evidence of organ dysfunction and hypoperfusion, acute alteration of mental status, elevated plasma lactate, unexplained metabolic acidosis (arterial ph <7.3), hypoxaemia, prolonged prothrombin time or decrease in platelet count >50% or ≤100 × 10^9^ litre^−1^, oliguria, and hypotension defined as systolic arterial pressure <90 mmHg or a decrease of >40 mmHg. Septic shock was defined as hypotension (<90/60 mmHg) in addition to sepsis syndrome persisting despite adequate fluid resuscitation and requiring intoropic support [6]. The SOFA score is composed of scores from six organ systems (respiratory R, cardiovascular C, hepatic H, coagulation Co, renal Re, and neurological N) graded from 0 to 4 according to the degree of dysfunction/failure. The aggregate score (total maximum SOFA score TMS) is calculated summing the worst scores for each of the organ systems (TMS_org_) during the ICU stay [7].

### 2.1. Estimation of Serum IL-8

Serum level of IL-8 was determined by quantitative sandwich enzyme immunoassay (R&D Systems, Inc., Minneapolis, MN, USA) according to the manufacturer's instructions. The intensity of the colour was measured at 490 nm for IL-8.

### 2.2. Estimation of IL-8 Gene Polymorphism

About 4 mL blood is withdrawn in EDTA-tubes and the genomic DNA was extracted using QIA amp DNA minikit from QIAGEN according to the manufacture instruction. Concentration of the extracted DNA was then measured by UV spectrophotometry (Pharmacia, Biotech, UK) at 260 and 280 nm and run on agarose gel electrophoresis 2% for detection of purity. Molecular detection of the (-251A/T) polymorphism in the IL-8 gene was achieved by restriction fragment length polymorphism typing. This included a combination of PCR amplification (Gene Amp PCR system 9700 from applied Biosystem) and digestion with restriction endonuclease MunI followed by gel electrophoretic analysis. Primer sequences for PCR were designed by Applied Biosystem. The primers used were forward: 5′-ATCTTGTTCTAACACCTGCCACTCT-3′ and reverse: 5′-TAAAATACTGAAGCTCCACAATTTGG-3′. PCR was carried out in a volume of 50 *μ*L containing 5 *μ*L (250) ng of genomic DNA, 25 *μ*L PCR Master Mix (containing 50 units/mL Taq DNA polymerase, supplied in a proprietary reaction buffer, pH 8.5, 400 *μ*M each: dATP, dGTP, dCTP, dTTP and 3 mM MgCl_2_), 5.0 *μ*L of each primer (10 *μ*M) and finally 10 *μ*L Nuclease-Free Water (Applied Biosystem, Foster city, CA, USA). The PCR conditions consisted of an initial denaturation step at 94°C, followed by 35 cycles of 94°C for 50 sec, 61°C for 1 min, 72°C for 55 seconds, and lastly a final elongation step at 72°C for 5 min were carried out. The amplification products were digested with Mun I restriction enzyme (Fermentas, Ottawa, ON, Canada) for 4 hours at 37°C, subjected to electrophoresis on 2% agarose gels stained with ethidium bromide. The generated PCR product of 121 bp was cleaved by restriction enzyme into fragments of 82 bp and 39 bp only if the A allele was present.

### 2.3. Statistical Analysis

Parametric data were analyzed using Student's *t*-test, while nonparametric data were analyzed using Mann-Whitney *U* and *χ* ^2^-tests. Data were presented as mean and standard deviation. A *P* value of <0.05 was considered significant. We calculated that we need 87 patients per group to have an 80% chance of detecting a 25% change in serum IL-8 at a 5% significance level, with a 2-sided significance level (nQuery Advisor, Version 5.0), so we included 90 patients per group.

## 3. Results

A total of 180 patients (97 men and 83 women) were included in the study. Ninety patients developed septic complication during ICU stay (*sepsis group*) in this group; eighteen patients developed septic shock and twenty eight patients developed severe sepsis. Ninety patients were critically ill without evidence of infectious organism (*SIRS group*). Forty-four patients died, fourteen of them were in septic shock and twenty were suffering from severe sepsis, while ten patients died in SIRS. There was no significant difference between the groups, except for SOFA score at ICU admission, the duration of the stay the ICU, and mortality rate which were higher in septic patients (Table 1).

The admission mean value of serum IL-8 is statistically significantly elevated in sepsis group which was 419.22 pg/mL with a range of (190–850 pg/mL) when compared to mean value in SIRS group which was 181.44 pg/mL with a range of (110–310 pg/mL) (*P* < 0.001) (Table 2).

The mortality rate is significantly higher in sepsis group, 34 patients died, while 56 patients survived. In SIRS group, 10 patients died, while 80 patients survived (*P* < 0.001) (Table 3).

In sepsis group, the mean value of serum IL-8 in survived patients was 297.679 ± 73.855 pg/mL which is significantly lower in comparison to the mean value in nonsurvived patients which is 619.412 ± 117.097 pg/mL (*P* < 0.001). In SIRS patients, the mean value of serum IL-8 in survived patients was 175.75 ± 36.380 pg/mL which is significantly lower in comparison to mean value in nonsurvived patients which is 227 ± 18.738 pg/mL (*P* < 0.001) (Table 4).

The incidence of gene mutation IL-8 (-251A/T) allele is similar in both groups. In sepsis group, 15 patients showed IL-8 (-251A/T) genetic polymorphism compared to 13 patients in SIRS group (*P* = 0.681) (Table 5).

In sepsis group, the mean value of serum IL-8 in patients with IL-8 (-251A/T) mutant allele was 694.333 ± 64.278 pg/mL which is significantly higher in comparison to mean value in patients with wild gene which is 364.2 ± 143.421 pg/mL (*P* < 0.001). In SIRS patients, the mean value of IL-8 in patients with IL-8 (-251A/T) mutant allele was 227.308 ± 43.235 pg/mL which was significantly higher in comparison to mean value in patients with wild gene which was 173.701 ± 31.762 pg/mL (*P* < 0.001) (Table 6).

A positive correlation of survival and IL-8 (-251A/T) mutant allele was detected in sepsis and SIRS patients respectively (*P* < 0.001) (Table 7).

**Figure 1:**
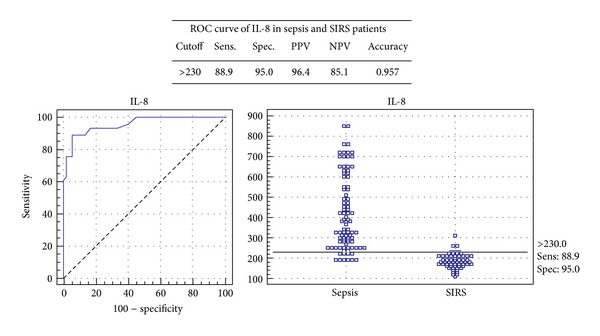
ROC curve of IL-8 in sepsis and SIRS patients.

The serum IL-8 distinguished sepsis from SIRS patients at a cut-off value of 230 pg/mL and demonstrated a sensitivity of 88.9%, specificity of 95%, and accuracy level of 0.957 with a positive predictive value of 96.4 and negative predictive value of 85.1 (Figure 1).

**Figure 2:**
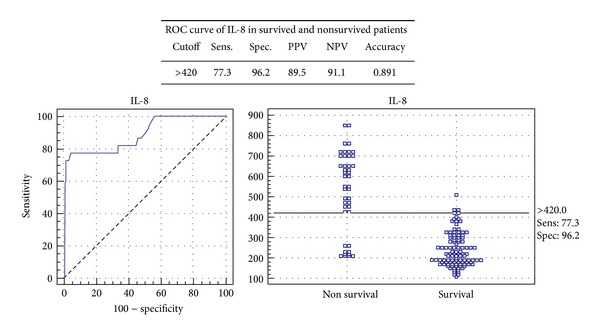
ROC curve of IL-8 in survived and nonsurvived patients.

The serum IL-8 in distinguished survived from non-survived patients in sepsis from SIRS patients at a cut-off value of 420 pg/mL and demonstrated a sensitivity of 77.3%, specificity of 96.2%, and accuracy level of 0.891 with a positive predictive value of 89.5 and negative predictive value of 91.1 (Figure 2).

**Figure 3:**
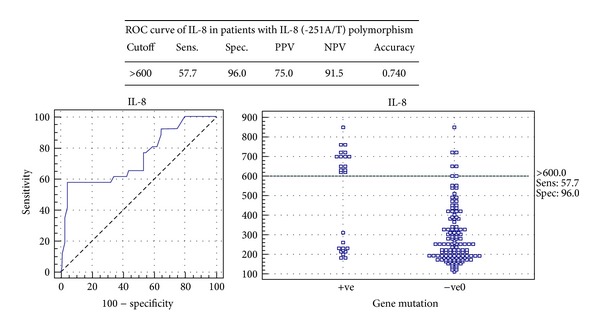
ROC curve of IL-8 in patients with IL-8 (-251A/T) polymorphism in the studied groups.

The serum IL-8 distinguished wild from patients with IL-8 (-251A/T) mutant allele at a cut-off value of 600 pg/mL and demonstrated a sensitivity of 57.7%, specificity of 96%, and accuracy level of 0.74 with a positive predictive value of 75 and negative predictive value of 91.5 (Figure 3).

## 4. Discussion

This novel study to our knowledge is the first to evaluate serum IL-8 and its genetic 251 polymorphism and the correlation of survival in critically ill septic and SIRS patients. Our study demonstrated that the admission mean value of serum IL-8 is significantly elevated in sepsis group; the mean value of serum IL-8 in non-survived patients is significantly higher than survived patients. The mean value of serum IL-8 in patients with IL-8 (-251A/T) mutant allele is significantly higher than patients with wild allele. A positive correlation of survival and IL-8 (-251A/T) mutant allele was detected in sepsis and SIRS patients, respectively. The serum IL-8 distinguished wild from patients with IL-8 (-251A/T) mutant allele in critically ill patients.

It has been long realized that not all individuals with a specific insult present with the same clinical pictures, nor do they have identical prognosis or response to treatment. The sequencing of the human genome and recognition of the degree of genetic variation that exists in the human population make it clear that an individual's genetic makeup is likely to have an impact on clinical manifestations as well as treatment and prognosis.

The response to infection is variable among different populations. Given the same medications, most patients will recover, while small portion may develop severe sepsis, multiple organ system failure, refractory hypotension, and may progress to death. This variability of patients' outcome had been attributed to a number of factors. Evidence is accumulating to support the theory that the genetic makeup of the host plays an important role in susceptibility to and development of sepsis, as well as its severity and prognosis. The genetic predisposition to the development of sepsis and to poor outcome from sepsis strongly exists [8].

Sequencing of the human genome had revealed that many genes are polymorphic, including some of the genes that have been implicated in the development of sepsis. The polymorphic gene is the gene in which comparison of the DNA sequence in multiple individuals shows differences at a frequency of 1%. These sites within the genes are called polymorphic sites. Some of these variations had been shown to influence the level and the activity of the resulting protein, thereby affecting cell function. The genes most likely to play a role in the susceptibility to and outcome from sepsis are mostly polymorphic genes that encode for protein products involved in the pathogenesis of sepsis [9].

The central event in the pathophysiology of sepsis is the release of proinflammatory cytokines in response to an inciting stimulus, such as a bacterial infection. Normally, these cytokines activate a variety of cellular and humoral systems that are responsible for eliminating this stimulus. However, an exaggerated inflammatory response leads to excessive production of these mediators resulting in an imbalance between the proinflammatory and the anti-inflammatory cytokines and consequently leads to the clinical manifestations of severe sepsis and multiple organ failure syndromes [10].

A number of the components involved in the body's response to bacterial infection and sepsis are encoded by polymorphic genes, including proteins involved with intracellular response to bacterial products such as interleukins (ILs). IL-8 is one of the pro-inflammatory cytokine; it belongs to the chemokine gene family of cytokines. It is produced by mononuclear phagocytes, endothelial cells, polymorphonuclear leucocytes, and a variety of mesothelial cell types in response to various stimuli. Its primary function is to activate and attract neutrophils to sites of inflammation [11].

Wong et al. [12] demonstrated increased IL-8 mRNA and protein in nonsurvivors of septic shock. Others demonstrated that initial IL-8 levels predict a high likelihood of survival in children and septic shock [13]. IL-8 is considered as a secondary pro-inflammatory cytokine with important chemotactic properties in systemic inflammation [14].

Miller et al. [15] demonstrated that IL-8 had been associated with inflammatory process in lung dysfunction in patients suffering from multiple organ dysfunction syndromes.

Biron et al. [16] demonstrated that serum level of IL-8 had been correlated with the course of sepsis in patients with lactacidemia. In addition, it had been reported that plasma IL-8 concentration was elevated in patients with sepsis and higher serum concentration correlate with mortality [17, 18].

Many studies have been indicated that polymorphisms in some of the genes are involved in the pathogenesis of sepsis. Genetic polymorphism in IL-8 at position-251 had been previously studied in various pathological conditions and this mutation had shown to be associated with increased IL-8 production; furthermore, this polymorphism was associated with diseases that include respiratory syncytial virus infection [5]. Baier et al. [19] studied the possibility of genetic association with septic complications and concluded that single nucleotide polymorphism of IL-6, IL-10, and Cd14 polymorphism may alter the risk of blood stream infection in ventilated very low birth weight infant.

Arnalich et al. [20] studied the polymorphism of IL-1 receptor agonist (IL-1Ra) gene on the outcome of severe sepsis and its effect on total production of interlukin-1 receptor antagonist protein and concluded that patients with polymorphism produced significantly lower level of IL-Ra which may contribute to higher mortality rate found in severe sepsis.

Schaaf et al. [21] studied the effect of interlukin-10, tumor necrosis factor alpha and lymphotoxin-alpha polymorphism regarding the development of septic shock in pneumococcal infection and concluded that patients with genetic predisposition for polymorphism may have higher risk of severe pneumococcal infection leading to septic shock. Wurfel et al. [22] studied the effect of single-nucleotide polymorphism (SNPs) affecting Toll-like receptor-mediated responses in healthy volunteer and found that genetic variation is associated with increased susceptibility to organ dysfunction, death, and gram-positive infection in sepsis. A strong association had been confirmed between TNF-*α* polymorphisms and the clinical presentation or outcome in patients with sepsis and septic shock [23, 24].

## 5. Conclusion

The admission mean value of serum IL-8 is significantly elevated in septic patients. Significant elevation in serum IL-8 in nonsurvived patients and patients with IL-8 (-251A/T) mutant alleles. A positive correlation of survival and IL-8 (-251A/T) mutant allele patients was detected. The serum IL-8 distinguished wild from patients with IL-8 (-251A/T) mutant allele critically ill patients.

## Figures and Tables

**Table 1:** Patient characteristics (mean and standard deviation).

	Sepsis group (*n* = 90)	Nonsepsis group (*n* = 90)
Age (years)	57.8 ± 13.4	54.8 ± 11.9
Sex ratio (M/F)	50/40	47/43
SOFA score mean (range)	14 (10–16)*	8 (4–12)
Duration of ICU stay	9.96 ± 3.007*	5.7 ± 2.27
Diagnosis		
Respiratory insufficiency due to:		
Bacterial infection	27	
ARDS	15	
COPD		25
Bronchial asthma		10
Pulmonary edema		11
Polytrauma	18	17
Orthopedic surgery	17	15
Thoracic surgery	13	12

*Statistically significant (*P* < 0.05).

**Table 2:** Comparison of serum IL-8 mean value in the studied groups.

Groups	IL-8		*t*-test	
	Range	Mean ± SD	*t*	*P* value
Sepsis	190.000–850.000	419.222* ± 181.824	12.139	<0.001
SIRS	110.000–310.000	181.444* ± 38.375		

*Statistically significant.

**Table 3:** Incidence of survival in studied groups.

Survival	Groups		
	Sepsis	SIRS	Total
Survival			
*N*	56	80	136
%	62.22	88.89	75.56
Nonsurvival			
*N*	34*	10	44
%	37.78	11.11	24.44
Total			
*N*	**90**	**90**	**180**
%	**100.00**	**100.00**	**100.00**
Chi-square			
*χ* ^2^		18.090	
*P* value		<0.001	

*Statistically significant.

**Table 4:** Concentration of serum IL-8 in survived and nonsurvived patients in the studied groups.

Groups	Survival	Nonsurvival	*t*-test	
	Mean ± SD	Mean ± SD	*t*	*P* value
IL-8				
Sepsis	297.679 ± 73.855	619.412 ± 117.097	−16.003	<0.001*
SIRS	175.750 ± 36.380	227.000 ± 18.738	−4.367	<0.001*

*Statistically significant.

**Table 5:** Incidence of gene mutation in the studied groups.

Gene mutation	Groups		
	Sepsis	SIRS	Total
Wild			
*N*	75	77	152
%	83.33	85.56	84.44
Mutant			
*N*	15	13	28
%	16.67	14.44	15.56
Total			
*N*	**90**	**90**	**180**
%	**100.00**	**100.00**	**100.00**
Chi-square			
*χ* ^2^		0.169	
*P* value		0.681	

**Table 6:** Concentration of serum IL-8 in patients with IL-8 (-251A/T) polymorphism in the studied groups.

Groups	Wild	Mutant	*t*-test	
	Mean ± SD	Mean ± SD	*t*	*P* value
IL-8				
Sepsis	364.200 ± 143.421	694.333 ± 64.278	−8.711	<0.001*
SIRS	173.701 ± 31.762	227.308 ± 43.235	−5.327	<0.001*

*Statistically significant.

**Table 7:** Correlation of survival and IL-8 (-251A/T) polymorphism in the studied groups.

Gene mutation	Survival		Nonsurvival		Total		Chi-square	
	*N*	%	*N*	%	*N*	%	*χ* ^2^	*P* value
Sepsis								
Normal	56	62.22	19	21.11	75	83.33	34.439	<0.001*
Poly	0	0.00	15	16.67	15	16.67		
Total	**56**	**62.22**	**34**	**37.78**	**90**	**100.00**		
SIRS								
Normal	73	81.11	4	4.44	77	85.56	13.396	<0.001*
Poly	7	7.78	6	6.67	13	14.44		
Total	**80**	**88.89**	**10**	**11.11**	**90**	**100.00**		

*Statistically significant.
